# First Trimester Phthalate Exposure and Infant Birth Weight in the Infant Development and Environment Study

**DOI:** 10.3390/ijerph13100945

**Published:** 2016-09-23

**Authors:** Sheela Sathyanarayana, Emily Barrett, Ruby Nguyen, Bruce Redmon, Wren Haaland, Shanna H. Swan

**Affiliations:** 1 Department of Pediatrics, University of Washington, 2001 W. 8th Ave, Seattle, WA 98121, USA; 2 Department of Environmental and Occupational Health Sciences, University of Washington, 2001 W. 8th Ave, Seattle, WA 98121, USA; 3 Seattle Children’s Research Institute, University of Washington, 2001 W. 8th Ave, Seattle, WA 98121, USA; wren.haaland@seattlechildrens.org; 4 Department of Obstetrics and Gynecology, University of Rochester, Rochester, NY 14642, USA; Emily_Barrett@URMC.Rochester.edu; 5 Division of Epidemiology and Community Health, University of Minnesota, Minneapolis, MN 55454, USA; nguyen@epi.umn.edu; 6 Department of Medicine, University of Minnesota, Minneapolis, MN 55455, USA; redmo001@umn.edu; 7 Icahn School of Medicine at Mount Sinai, Department of Preventive Medicine, New York, NY 10029, USA; shanna.swan@mssm.edu

**Keywords:** birth weight, phthalate, preterm, fetal growth, first trimester

## Abstract

Phthalate exposure is widespread among pregnant women but whether it is related to fetal growth and birth weight remains to be determined. We examined whether first trimester prenatal phthalate exposure was associated with birth weight in a pregnancy cohort study. We recruited first trimester pregnant women from 2010–2012 from four centers and analyzed mother/infant dyads who had complete urinary phthalate and birth record data (*N* = 753). We conducted multiple linear regression to examine if prenatal log specific gravity adjusted urinary phthalate exposure was related to birthweight in term and preterm (≤37 weeks) infants, stratified by sex. We observed a significant association between mono carboxy-isononyl phthalate (MCOP) exposure and increased birthweight in term males, 0.13 kg (95% CI 0.03, 0.23). In preterm infants, we observed a 0.49 kg (95% CI 0.09, 0.89) increase in birthweight in relation to a one log unit change in the sum of di-ethylhexyl phthalate (DEHP) metabolite concentrations in females (*N* = 33). In summary, we observed few associations between prenatal phthalate exposure and birthweight. Positive associations may be attributable to unresolved confounding in term infants and limited sample size in preterm infants.

## 1. Introduction

Low birth weight and intrauterine growth retardation are significant risk factors for future morbidity including obesity and poor cognitive function [1], yet identification of modifiable environmental exposures that influence fetal growth remains to be determined. Phthalates are a class of man-made chemicals that are ubiquitous in the general population [2]. Pregnant women have widespread exposure from contact with plastic products, personal care products, contaminated foods, and dust [3]. Phthalates cross the placenta and can impact fetal development leading to adverse reproductive outcomes [3] via hormone mediation, oxidative stress, and peroxisome proliferator activated receptor (PPAR) pathways [4,5]. These pathways may also be important for fetal growth, but it is unclear whether first trimester phthalate exposures are associated with birth weight outcomes.

Some rodent toxicologic studies indicate that gestational di-ethyl hexyl phthalate (DEHP) exposure leads to reduced birth weight in offspring [6,7]. In humans, results are conflicting with regard to the relationship between prenatal phthalate exposures and birth weight. Chinese and a combined European birth cohort study reported that prenatal DEHP exposure is associated with growth restriction and reduced birth weight [8,9,10] while findings in French and New York City birth cohorts were null [11,12]. A Spanish study of fetal ultrasound measurements found minor associations between prenatal monobenzyl (MBzP) and monobutyl (MBP) phthalate exposures and femur length, head circumference, and birth weight in a sex-specific manner [13]. The majority of these studies measured phthalate concentrations in the second or third trimester of pregnancy, and few reported significant findings with non-DEHP phthalate compounds. Some reported differences in associations among males versus females but results were conflicting. The disparate finding across studies may be due to differences in exposure concentrations, sample size, study design, and timing of exposure during pregnancy.

Our aim was to assess prenatal first trimester phthalate exposure in relation to birth weight among term and preterm infants in a prospective multi-center pregnancy cohort study, The Infant Development and the Environment Study (TIDES). We hypothesized that first trimester DEHP exposures would be inversely associated with birth weight outcomes.

## 2. Materials and Methods

### 2.1. Study Population

We summarize recruitment of the TIDES cohort and its characteristics briefly; details are discussed elsewhere in Barrett et al. 2014 [14]. TIDES recruited first trimester pregnant mothers from four study centers at the University of California, San Francisco (UCSF), University of Minnesota (UMN), University of Rochester Medical Center (URMC), and Seattle Children’s Hospital/University of Washington (SCH/UW) from 2010–2012. Eligibility criteria included: less than 13 weeks pregnant, singleton pregnancy, English speaking, age 18 or over, no serious threat to the pregnancy, and plans to deliver at a study hospital. Interested women who met eligibility criteria signed an informed consent for themselves and their infant, and were then enrolled in the study. All study centers received human subjects’ approval for conducting TIDES procedures. Study participants completed a questionnaire on demographics and gave a urine sample in each trimester of pregnancy. We report on 753 women who completed questionnaires and delivered a live infant. We recorded birth weight from the official medical record at birth from each institution. We used the first available ultrasound in the medical record to calculate gestational age. If no ultrasound was available, we used the physician’s estimate of gestational age at birth.

### 2.2. Phthalate Assessment

First trimester urine was collected in sterile and phthalate-free polypropylene specimen cups during initial recruitment visits. Urine was transferred into 5 individual 2 mL cryovials, and stored in freezers at <−80 °C. We measured specific gravity using a handheld refractometer at the time of urine collection, which was calibrated with deionized water before each measurement. Specific gravity is used to correct for urinary dilution. Phthalate metabolite concentrations were analyzed at two different sites. Three hundred subject samples for mothers of female infants were analyzed at the Environmental Health Laboratory at the University of Washington (UW). Per a modified version of the CDC method 6306.03, glucuronidated phthalate monoesters underwent enzymatic deconjugation, followed by online-solid phase extraction (SPE) coupled with reversed high performance liquid chromatography-electrospray ionization-tandem mass spectrometry (HPLC-ESI-MS/MS) to quantify the simple monoesters in urine [15]. An additional 369 subject samples for mothers of male infants were analyzed by the Division of Laboratory Sciences, National Center for Environmental Health, Center for Disease Control and Prevention (CDC). At the CDC, urine samples were analyzed using a modified method described in Silva et al. 2007 that involved the enzymatic deconjugation of the phthalate metabolites from their glucuronidated form, automated on-line sold phase extraction, separation with high performance liquid chromatography and detection by isotope-dilution tandem mass spectrometry [16]. Process and instrument blanks as well as field blanks were run in each lab for quality assurance of analytical and sampling procedures. For the field blank collection, deionized water was purchased, poured into phthalate-free urine cups and transferred with disposable pipettes to 5 mL cryovials. These blanks were then interspersed with subject samples to be shipped to laboratories. Ten urine samples were also analyzed at both UW and the CDC for comparison and showed values that were within 10% of one another for MEP, MBP, and MBzP. For the DEHP metabolites and MCPP, values differed between labs. Results are presented for male and female infants separately and together in the results section.

The limit of detection (LOD) of metabolites was between 0.2 and 2.0 ng/mL for the UW samples and 0.2 and 0.6 ng/mL for the CDC samples. For concentrations below the LOD, a value equal to each sample’s specific LOD divided by the square root of 2 was used [17]. All urinary phthalate metabolite levels were adjusted for dilution using specific gravity measurements and logarithmically transformed to normalize distributions. To calculate the molar sum of the DEHP metabolites, mono-2-ethylhexyl phthalate (MEHP), mono-2-ethyl-5-hydroxy-hexyl phthalate (MEHHP), mono-2-ethyl-5-oxy-hexyl phthalate (MEOHP) and mono-2-ethyl-5-carboxypentyl phthalate (MECPP) were divided by their molecular weights and added (∑DEHP metabolites = (MEHP/278) + (MEHHP/294) + (MEOHP/292) + (MECPP/308)) × 1000). Other metabolites measured included mono-ethyl phthalate (MEP), MBzP, MBP, mono-3-carboxypropyl (MCPP), and mono (carboxy-isononyl) phthalate (MCOP).

### 2.3. Statistical Analysis

We examined the distributions of demographic characteristics and specific gravity adjusted phthalate concentrations. All phthalate concentrations were log transformed for analysis because of skewedness. Gestational age at birth is an important factor to account for in birth weight analyses, and traditional methods of covariate adjustment in regression models may not be appropriate [18,19,20]. Therefore, we constructed birth weight for gestational age z-scores as our outcome variable using methods put forth by Olsen et al in 2010 and used values in Talge et al. 2014 to update previous curve calculations [18,21]. We compared results of this constructed variable to that of crude birthweight and gestational age and did not see significant differences. Therefore, we present models for multiple regression with crude birthweight as the primary outcome variable. We also present models without adjustment for gestational age (our primary model) and those with gestational age (sensitivity analysis) due to the uncertainty of how to account for gestational age in the published literature. Several variables were considered for confounding in the analysis. After reviewing the literature, we decided a priori to adjust for study center, smoking during pregnancy, race/ethnicity, and parity in all analyses given their well-established relationship to the outcomes of interest. We also examined time of day of urine collection, maternal age, education status, pre-pregnancy BMI, and income to see if point estimates changed appreciably or if they improved the precision of the model. We examined infant sex in stratified analyses given the literature noting differential growth patterns in males versus females. We present multiple linear regression t in term and preterm infants (≤37 weeks) separately and together because growth patterns can differ appreciably between the two groups even accounting for gestational age. In order to assess for highly influential observations, graphical examination of the residuals using Stata’s qnorm plot showed that in the male pre-term group, one point in particular was quite a distance from the line indicating normality. This point represented the individual with the heaviest birthweight. Therefore, we excluded this infant from the analysis.

### 2.4. Results

The majority of women were between 20–40 years old (93%), white (66%), and had a college degree (74%) (Table 1). A small percentage (7%) reported smoking anytime during pregnancy. Preterm infants comprised 10% of the total sample, and the Universities of Rochester and Minnesota had a higher percentage of preterm births compared to the Universities of California, San Francisco (Table 1). Mean birth weight in term infants was 3440 g (SD 0.48) and 2600 g (SD 0.67) in preterm infants. The percentage of samples above the limit of detection ranged from 66%–99%, and the distribution of phthalate concentrations is shown in Table 2 and also reported in previous publications in further detail [19]. Correlations between the DEHP metabolites (MEHP, MEOHP, MEHHP, and MECPP) were between 0.6–0.9 while the correlations between all other metabolites ranged in absolute magnitude from 0–<0.6 (results not shown).

In male, term infants, MCOP was significantly associated with a 0.13 kg increase (95% CI 0.03, 0.23) in birth weight in relation to one log unit change of MCOP (Table 3), and this result persisted in models adjusting for gestational age (Table 4). No significant relationships were observed in term female infants. In preterm female infants, the log sum of DEHP metabolites was significantly associated with a 0.49 kg (95% CI 0.09, 0.89) increase in birth weight, and all individual DEHP metabolites were also significantly associated with birth weight, and these relationship persisted in models adjusting for gestational age except for MEHP which was not statistically significant (Table 3). In our sensitivity analysis adjusted for gestational age, we observed a −0.36 kg (−0.68, −0.05) decrease in birth weight relation to a one log unit change in MBP in male preterm infants (Table 4). Of note, the direction of the coefficients for the analyses was often in the opposite direction for males versus females.

## 3. Discussion

The hypothesis that DEHP exposure would be inversely related to birthweight outcomes was not supported by our findings. In contrast, we observed positive associations in preterm infants. Possible explanations include differences in timing of exposure during pregnancy and exposure concentrations changing over time. Our study is one of two to examine first trimester exposures in relation to birth weight outcomes [13]. Our findings in term infants are similar to those in Wolff et al. who examined third trimester urinary concentrations in in New York City and different from European and Chinese cohort studies [8,9,11]. Compared with other birth cohort studies [9,11], urinary phthalate concentrations were lower in TIDES but this could be due to differences in measurement technique or changes in exposure over time. Our reported exposure concentrations were similar or slightly lower to those of females in NHANES from 2011–2012 [2], and these concentrations are lower than other birth cohort studies. This finding likely reflects that DEHP use has decreased in the past decade while di-isononyl phthalate (DINP) use has increased [3]. Of note, we report measureable concentrations of the DINP metabolites, MCOP [3]. Our study population is geographically diverse but not racially or ethnically diverse compared to the general population, and minority populations tend to bear a larger burden of environmental exposures. Environmental sources of phthalate exposures in the general population include personal care products for the low molecular weight phthalates such as diethyl phthalate and dibutyl phthalate and food and dust contamination for the high molecular weight phthalates such as DEHP and DINP [5].

We examined males and females separately based on differential growth patterns between the sexes. We observed a suggestion of sex-specific effects with DEHP being positively associated with birth weight in female, preterm infants, and MBP inversely associated with birth weight in male, preterm infants. Other birth cohort studies noted sex specific effects with respect to phthalate exposures, but findings differed with respect to phthalate and timing of exposure [10,13]. Several studies of other health endpoints such as neurobehavior and anogenital distance report sex-specific impacts of phthalate exposure likely due to phthalate related hormonal impacts differentially affecting each sex during fetal development [22,23]. Our findings should be confirmed in a larger sample size of premature infants. Of note, the direction of the point estimates often differed among males and females in term infants, but given the lack of statistical significance, we cannot make conclusions based on these findings.

Other birth cohort studies differed from ours based on timing of exposure as well as matrix of measurement. We examined first trimester exposures while cohorts in New York, China, and Europe measured concentrations in the second or third trimesters, and serum or cord blood samples were used for phthalate measurements in the European cohorts [8,9,10,11]. One study examined an average concentration from first and third trimester spot measurements but did not look at trimester specific effects [13]. The period of major growth for a fetus is the third trimester, but it may be that first trimester exposures program growth processes over the course of the pregnancy. One of the studies used a nested case-control design to compare small-for-gestational age or low birth weight infants to controls, [9] which is good for rare outcomes.

Primary predictors of birth weight included socio-economic factors and maternal infections [24]. While the latter are modifiable in pregnancy with good prenatal care, other SES factors are not easily changeable. Environmental endocrine disrupting chemical exposures are thought to play a role in fetal development because they impact hormonal processes and oxidative stress, both of which are known to affect growth and birth outcomes. Recent studies report that prenatal phthalate exposure may affect time of gestation and preterm birth outcomes through a PPAR-γ oxidative stress mechanism [25]. While PPAR-γ is known to be associated with growth, obesity and other metabolic outcomes, the finding of preterm birth may reflect a maternal effect and not one on the fetus and therefore, fetal growth would not be affected. In addition, while rodent models show that phthalates affect liver PPAR-γ, human liver cells do not exhibit the same response [26]. At this time, other potential hypotheses for effects on fetal growth remain to be determined.

Strengths of our study include the prospective study design that examined women from four different locations and centers. Birth weight was recorded directly from the medical record, and all lab personnel performing phthalate analyses were blinded to fetal outcomes. We obtained one spot urine sample in the first trimester of pregnancy. Concentrations can be variable over pregnancy, but one major predictor of variability is time of day of urine collection because ingestion of meals is related to exposure concentrations [27]. We examined time of day in our analyses that did not change point estimate outcomes and was therefore not included in final models. We performed laboratory phthalate analysis at two different labs, and the concordance between 10 samples differed according to metabolite. Samples from mothers of sons were performed at the CDC while samples from mothers of daughters were performed at UW. Because we chose a priori to examine sex in a stratified manner, we do not feel that the values from the two different labs affected our results. Another limitation is the large number of comparisons performed which could lead to false associations but given that the DEHP metabolites have a high degree of collinearity, multiple comparisons analysis was not performed.

Our analysis found little evidence to support a relationship between first trimester prenatal phthalate exposure and birth weight; and while there is a suggestion of an effect in preterm infants in a sex-specific manner, the sample size is too small to draw conclusions. Future studies should examine first trimester exposures in relation to birth weight in larger sample sizes that can accurately assess impacts in premature infants.

## 4. Conclusions

In summary, we observed few associations between prenatal phthalate exposure and birthweight. First trimester prenatal phthalate MCOP exposure was associated with increased birthweight in male infants only. We did observe a positive effect for DEHP in preterm, female infants but our findings should be interpreted with caution. Positive associations may be attributable to unresolved confounding in term infants or limited sample size in preterm infants.

## Figures and Tables

**Table 1:** Demographic characteristics of 753 mom infant dyads in TIDES.

Characteristic	Total *n* (%)	Term *n* (%)	Preterm *n* (%)
Study Center			
UCSF	187 (25)	173 (25)	14 (19)
UMN	202 (27)	180 (27)	22 (30)
URMC	212 (28)	186 (27)	26 (35)
UW	152 (20)	140 (21)	12 (16)
Maternal Age (years)			
<20–<30	291 (39)	260 (38)	31 (43)
30–<40	426 (57)	393 (58)	33 (45)
>40	32 (4)	23 (3)	9 (12)
Pre-Pregnancy BMI			
20–<24.9	435 (58)	405 (60)	30 (41)
25–<29.9	164 (22)	144 (21)	20 (28)
>30	145 (19)	123 (18)	22 (31)
Race/Ethnicity			
NH White	496 (66)	446 (66)	50 (68)
NH Black	86 (11)	75 (11)	11 (15)
NH Asian	48 (6)	46 (7)	2 (3)
Hispanic	67 (9)	61 (9)	6 (8)
Other/Mixed/Unknown	52 (7)	48 (7)	4 (5)
Education			
≤High school/Some college	191 (26)	173 (26)	18 (25)
College/post graduate	554 (74)	499 (74)	55 (75)
Any Smoking During Pregnancy			
Yes	54 (7)	50 (7)	4 (6)
No	695 (93)	627 (93)	68 (94)
Parity			
Nulliparous	276 (38)	252 (38)	24 (35)
Parous	451 (62)	406 (62)	45 (65)
Gestatational Age at Birth (weeks)			
25–≤37	74 (10)	N/A	74 (100)
>37	679 (90)	679 (100)	N/A
Income			
<$25,000	173 (24)	149 (23)	24 (33)
$25,000–74,999	197 (27)	185 (28)	12 (17)
≥$75,000	356 (49)	320 (49)	36 (50)
Infant Sex			
Male	369 (49)	331 (49)	38 (51)
Female	384 (51)	348 (51)	36 (49)
Infant Birthweight, kg (mean (SD))	3.35 (0.6)	3.44 (0.5)	2.60 (0.7)

**Table 2:** Percentiles of specific gravity adjusted first trimester urinary phthalate concentrations in TIDES.

Phthalate Metabolite	% > LOD	25%	Median	75%
MBP	92	4.83	8.45	14.05
MBzP	87	2.05	4.00	8.56
MEP	99	13.69	31.32	81.93
MCPP	75	1.03	2.05	4.79
MCNP	96	1.45	2.18	4.41
MCOP	100	8.12	15.20	44.04
MEHP	66	1.37	2.49	4.35
MEHHP	97	4.35	7.56	12.77
MEOHP	97	3.13	5.44	8.70
MECPP	98	5.90	9.51	15.95

**Table 3:** Birthweight (kg) in relation to log specific gravity adjusted prenatal phthalate exposure ^a^.

	Male Infants			Female Infants		
	All Births *n* = 351	Term Birth *n* = 315	Preterm Birth *n* = 35	All Births *n* = 356	Term Birth *n* = 323	Preterm Birth *n* = 33
MBP	−0.11 (−0.26, 0.04)	−0.01 (−0.15, 0.13)	−0.27 (−0.80, 0.25)	0.14 (−0.02, 0.31)	0.14 (−0.01, 0.29)	0.35 (−0.32, 1.03)
MBzP	0.00 (−0.13, 0.14)	−0.05 (−0.17, 0.07)	0.02 (−0.53, 0.57)	0.04 (−0.10, 0.18)	0.09 (−0.04, 0.22)	0.35 (−0.04, 0.74)
MCPP	0.04 (−0.07, 0.16)	0.07 (−0.03, 0.17)	−0.14 (−0.53, 0.24)	0.04 (−0.06, 0.14)	0.02 (−0.07, 0.11)	0.15 (−0.16, 0.46)
MEP	−0.04 (−0.15, 0.06)	0.00 (−0.09, 0.10)	−0.11 (−0.47, 0.25)	0.01 (−0.09, 0.10)	−0.01 (−0.10, 0.08)	0.15 (−0.16, 0.45)
MEHP	−0.10 (−0.25, 0.06)	−0.06 (−0.20, 0.08)	−0.29 (−0.80, 0.21)	0.13 (−0.01, 0.26)	0.09 (−0.04, 0.22)	0.46 * (0.09, 0.83)
MEHHP	−0.09 (−0.23, 0.05)	−0.05 (−0.18, 0.08)	−0.01 (−0.50, 0.47)	0.15 * (0.00, 0.29)	0.08 (−0.06, 0.22)	0.43 * (0.04, 0.82)
MEOHP	−0.12 (−0.26, 0.03)	−0.07 (−0.20, 0.06)	−0.03 (−0.53, 0.48)	0.11 (−0.04, 0.25)	0.04 (−0.11, 0.18)	0.51 * (0.11, 0.90)
MECPP	−0.07 (−0.24, 0.09)	−0.01 (−0.16, 0.14)	0.01 (−0.58, 0.61)	0.14 (−0.00, 0.28)	0.04 (−0.10, 0.18)	0.47 * (0.06, 0.88)
MCOP	0.07 (−0.04, 0.18)	0.13 * (0.03, 0.23)	−0.26 (−0.66, 0.13)	−0.10 (−0.34, 0.15)	−0.08 (−0.30, 0.14)	N/A
Sum DEHP	−0.10 (−0.25, 0.06)	−0.04 (−0.19, 0.10)	−0.04 (−0.61, 0.53)	0.15 (−0.00, 0.30)	0.07 (−0.08, 0.22)	0.49 * (0.09, 0.89)

Note: MCOP measurements are not available for the majority of female infants; excludes one male preterm outlier. ^a^ Adjusted for race, smoking during pregnancy, study center, parity, income. * *p* < 0.05.

**Table 4:** Birthweight (kg) in relation to log specific gravity adjusted prenatal phthalate exposure adjusted for gestational age at birth ^a^.

	Male Infants			Female Infants		
	All Births *n* = 351	Term Birth *n* = 315	Preterm Birth *n* = 35	All Births *n* = 356	Term Birth *n* = 323	Preterm Birth *n* = 33
MBP	−0.01 (−0.13, 0.11)	0.03 (−0.10, 0.16)	−0.36 * (−0.68, −0.05)	0.09 (−0.05, 0.22)	0.10 (−0.04, 0.24)	0.29 (−0.27, 0.85)
MBzP	−0.00 (−0.11, 0.11)	−0.00 (−0.12, 0.11)	−0.12 (−0.49, 0.24)	0.07 (−0.05, 0.19)	0.09 (−0.04, 0.21)	0.16 (−0.22, 0.53)
MCPP	0.06 (−0.03, 0.15)	0.07 (−0.02, 0.17)	−0.05 (−0.30, 0.21)	0.02 (−0.07, 0.10)	0.01 (−0.08, 0.10)	0.04 (−0.24, 0.31)
MEP	−0.00 (−0.09, 0.08)	0.02 (−0.07, 0.11)	−0.15 (−0.38, 0.08)	−0.01 (−0.09, 0.07)	−0.02 (−0.11, 0.06)	0.10 (−0.16, 0.35)
MEHP	−0.02 (−0.14, 0.10)	−0.01 (−0.14, 0.12)	−0.21 (−0.54, 0.12)	0.13 * (0.01, 0.24)	0.07 (−0.05, 0.19)	0.30 (−0.04, 0.65)
MEHHP	−0.03 (−0.15, 0.08)	−0.02 (−0.14, 0.10)	−0.08 (−0.39, 0.24)	0.15 * (0.03, 0.27)	0.08 (−0.06, 0.21)	0.32 (−0.02, 0.66)
MEOHP	−0.05 (−0.17, 0.07)	−0.04 (−0.16, 0.09)	−0.10 (−0.43, 0.23)	0.13 * (0.00, 0.25)	0.04 (−0.10, 0.17)	0.38 * (0.03, 0.73)
MECPP	−0.01 (−0.14, 0.12)	0.02 (−0.12, 0.15)	−0.09 (−0.48 , 0.30)	0.14 * (0.02, 0.26)	0.04 (−0.09, 0.17)	0.40 * (0.06, 0.74)
MCOP	0.10 * (0.01, 0.18)	0.12 * (0.03, 0.21)	−0.17 (−0.43, 0.10)	−0.01 (−0.20, 0.18)	−0.00 (−0.20, 0.19)	N/A
Sum DEHP	−0.03 (−0.16, 0.10)	−0.01 (−0.14, 0.13)	−0.11 (−0.48, 0.26)	0.16 * (0.03, 0.29)	0.06 (−0.08, 0.21)	0.37 * (0.02, 0.72)

Note: MCOP measurements are not available for the majority of female infants; excludes one male preterm outlier. ^a^ Also adjusted for race, smoking during pregnancy, study center, parity, income. * *p* < 0.05.
